# The effects of resistance based post-activation performance enhancement on reaction time and change of direction in basketball players

**DOI:** 10.1371/journal.pone.0320437

**Published:** 2025-03-26

**Authors:** Sümeyye Genç, Ahmet Rahmi Günay, Erkan Günay

**Affiliations:** 1 Institute of Health Sciences, Dokuz Eylul University, İzmir, Turkiye; 2 Faculty of Sport Sciences, Muğla Sıtkı Koçman University, Kötekli, Turkiye; 3 Faculty of Sport Sciences, Manisa Celal Bayar University, Manisa, Turkiye; NED University of Engineering and Technology, PAKISTAN

## Abstract

The aim of this study was to investigate the effect of post-activation performance enhancement (PAPE) intervention with 80% one repetition maximum (1RM) resistance on change of direction (COD) and reaction time (RT) in basketball players. This study sixteen male basketball players (mean age: 20.25 years, height: 1.88 m, weight: 80.75 kg, training age: 10.12 years) were included. For this study, participants attended 3 experimental sessions in the laboratory. Firstly, anthropometric measurements of the participants were taken, then RT and COD were familiarized respectively, and then 1RMs were determined. Then, the participants randomly completed the first and second sessions. In the first session, a 20-minute standard warm-up (Wup) was performed. After the participants rested passively for 3 minutes after the Wup, RT and COD tests were measured at 1-minute intervals, respectively. The results obtained were considered as the control condition. In the second session, participants rested passively for 3 minutes after performing the PAPE (80% of 1RM – 5 rep) protocol. After the rest period, participants performed RT and COD with a 1-minute interval, respectively. The data were analyzed separately for RT (visual, auditory, and mixed) and COD test results in terms of Wup and Wup+PAPE. At least 48 hours of rest was allowed between the first and second sessions to ensure that fatigue from the previous test session did not affect the results. Wilcoxon test results showed that PAPE significantly reduced visual RT (p < .005), mixed RT (p < 0.013), and COD (p < 0.001), but not auditory RT (p < 0.068). The findings showed that PAPE is an effective method to improve COD and RT performance in sports such as basketball, where success is achieved through fast-paced play.

## Introduction

In the literature, researchers emphasize that acute performance improvement can be achieved with warm-up (Wup) activities [1]. In this sense, a Wup that includes post-activation performance enhancement (PAPE) can be an effective strategy compared to a traditional Wup in sports disciplines. PAPE is described as a voluntary muscle contraction that results in an increase in muscle strength that is primarily caused by the phosphorylation of myosin light chain in type II muscle fibers [2]. PAPE intervention with resistance exercise a few minutes before competition involves changes in the neuromuscular system that may affect cognitive performance [3] as well as acute improvement [4].

All movements that affect the outcome in competition involve cognitive functions such as reaction time (RT) [5] and neuromuscular processes such as chance of direction (COD) [6]. These two important characteristics are at the forefront of performance in tight and fast-paced games such as basketball [7]. Therefore, players must demonstrate a high level of RT [8] and COD [9] performance that allows them to move effectively in response to a sudden external stimulus (e.g., passing, approaching an opponent) in planned or unplanned situations within the basketball game. The development of motor skills such as acceleration, deceleration and COD, and cognitive skills such as decision-making, which contribute significantly to the achievement of this performance, and the evaluation of the results are necessary to understand the physical and cognitive needs of athletes [10].

The effects of acute resistance exercise on cognitive performance have received increasing attention, and results have suggested that resistance exercise has the potential to affect cognitive performance [11,12]. It is speculated that the increase in arousal is responsible for this effect on cognitive performance. It is suggested that this increase in arousal is due to exercise-induced release of norepinephrine and subsequent release of dopamine, which increases extracellular catecholamines in brain regions responsible for cognition [13].

COD, which is an important component of performance, is defined as the ability to change direction quickly, change direction quickly and accurately, or the ability of the limbs to move and change direction quickly [14]. It has been reported in the literature that resistance training may be a good performance variable for COD in basketball players; however, despite the interest in resistance performance [15], there are only a limited number of studies created with the PAPE intervention that have examined its effect on COD performance [16].

In the literature, the effects of resistance-based PAPE on factors such as sprinting [17–19], jumping [20–22], and maximal voluntary contraction [23,24], which can be related to basketball performance, have been frequently examined. In the basketball game, the ability to generate quick responses to visual and auditory RT inputs and COD tasks in which acceleration and deceleration phases are frequently used is exposed. For this reason, there is a need to focus on the effects of resistance-based PAPE on COD and RT components. Therefore, the aim of this study was to determine whether PAPE intervention with 80% of 1RM resistance has an effect on COD and visual, auditory, and mixed RT in basketball players. Two hypotheses were proposed: a) resistance-based PAPE intervention positively affects COD performances, including acceleration and deceleration tasks b) resistance-based PAPE positively affects RT performance.

## Methods

### Experimental approach to the problem

**Fig 1:**
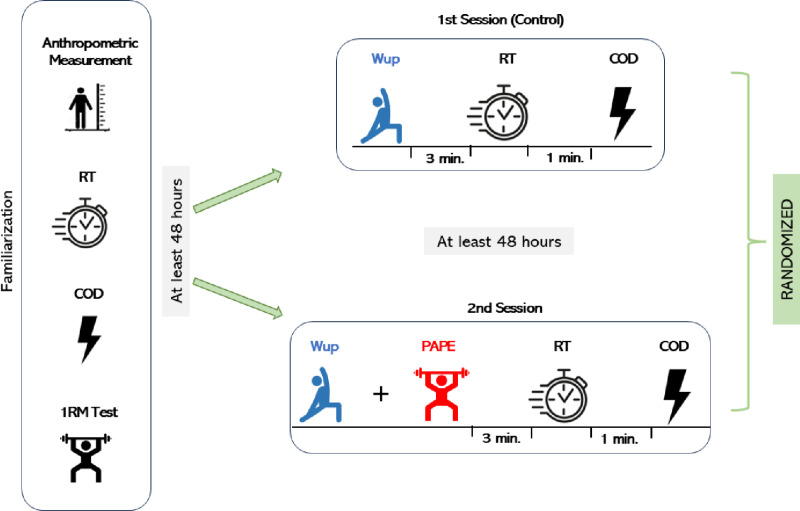
Experimental Design. Wup: Warm-up, RT: Reaction Time, COD: Change of Direction, 1RM: One Repetition Maximum, PAPE: Post-activation Performance Enhancement.

For this study, participants attended 3 experimental sessions in the laboratory. All trials were performed at the same time of the day (10:00 am - 12:00 pm). Firstly, anthropometric measurements of the participants were taken, then RT and COD were familiarized respectively, and then 1RMs were determined. Then, the participants randomly completed the first and second sessions. In the first session, a 20-minute standard Wup was performed. After the participants rested passively for 3 minutes after the Wup, RT and COD tests were measured at 1-minute intervals, respectively. The results obtained were considered as the control condition. In the second session, participants rested passively for 3 minutes after performing the PAPE protocol [25]. After the rest period, participants performed RT and COD with a 1-minute interval, respectively. At least 48 hours of rest was allowed between the first and second session to ensure that fatigue from the previous test session did not affect the results (Fig 1). The recruitment period for participants was conducted between 05/04/2022 and 08/05/2022.

### Participants

The study included 16 male players who were active in the university league basketball team. The sample size was determined previously by G * Power software (v. 3.1.9.4), assuming α =  0.05, β =  0.95 and effect size =  1.20 based on a previous study [26]. Before starting the tests of the study, the players were prohibited from intense physical activities for 3 days before the study and caffeine consumption for 24 hours before the study. The study protocol is in accordance with the latest version of the Declaration of Helsinki. Participants were informed about the potential risks and experimental designs of the study before all tests, and their written informed consent was obtained; since only adults were included in the study, parental or guardian consent was not required. This study received ethical approval from Muğla Sıtkı Koçman University Medical and Health Sciences Ethics Committee (210064/35).

### Anthropometric data collection

In the determination of height and body weight, a Seca (Germany) electronic height and weight meter with 0.01 kg and 1 mm of sensitivity was used. Players participated in the test with bare feet, shorts, and t-shirts. Height “m” and body weight “kg” were used to record data [27].

### Standardized warm-up protocol

The 20-minute standardized warm-up protocol consisted of standardized warm-up and dynamic stretching phases. In the general warm-up, mild to moderate tempo running and dynamic stretching, basketball-specific drills were applied. The dynamic stretching phase was performed at a smooth pace, with a total of 6 movements (standing quadriceps stretching, standing knee pull, hip internal rotation, hip external rotation, standing hamstring stretching, standing toe touch) in 2 sets in a 12-meter area. Sprinting and movements involving high power output and high intensities that may occur due to potential potentiation effects were avoided [28].

### One repetition maximum test

We used the participants’ previous training experiences to determine the initial load of the 1RM test, and they made their first attempts with a load that they could lift 4 repetitions in one set. If the first load yielded more or less than 4 repetitions, we changed the applied load and took a 5-minute break. We continued this procedure until we performed four lifts using the correct technique. We then applied 4 RM a second time for verification purposes, following a 5-minute rest. If the number of repetitions increased in the second application, the load increased again. In the lifting technique, we ensured that the participant started with the bar at shoulder level, their feet shoulder-width apart, and their knees in a straight position. The participant maintained a 90° knee angle and applied a rhythm of one-second eccentric, one-second transition, and one-second concentric phases during the movement [29].

The Epley formula (1RM =  [weight lifted x number of reps x 0.0333] +  weight lifted) was used to calculate the estimated 1RM of the participants [30].

### Post-activation performance enhancement protocol

For the PAPE protocol, each participant performed 5 repetitions of back squats (1 second eccentric and 1 second concentric movements at 90° knee angle, 1 second rest between each repetition) with 80% of the 1RM resistance for the PAPE intervention [31,32] after 20 minutes of standard Wup. A 20-kg bar and free weights were used during the back squat. Google metronome (60 bpm) was used to control the speed of movement. A height-adjustable seat was placed behind the participant to standardize the back squat depth during the repetition of the movement.

### Reaction time measurement

A Newtest 1000 (Model 90220 Finland) reaction timer was used to determine the RT performance of the participants. The Newtest 1000 was placed on the table 10 cm away from the participant. [33] The test was performed in a lighted, quiet environment, with the participant in a comfortable body position to easily perceive the test panel, standing, and with the dominant hand on the table. When an audible or lighted stimulus was given with the “ready” command, the participant was asked to press the keys as soon as possible according to the stimulus. For each test, 5 (15 in total) random stimuli were presented as visual (light), auditory (sound), and mixed (light or sound), and the mean of the 5 scores was recorded as RT in milliseconds [34].

### Change of direction test

The Illinois Agility Test is a highly ergonomic and effective test widely used to determine COD performance. The test track is 5 meters wide and 10 meters long. In the center, there are a total of 4 pins spaced 3.3 meters apart. The Newtest 1000 photocell devices (Model 90220 Finland) are placed at the start and end gates on the long side of the test area for time detection. The participant was lying face down at the starting point with his or her hands at shoulder level. On the researchers’ command “Go,” the participant stood up as fast as possible and ran around the track in the indicated direction, trying not to touch the cones. He then ran towards the cone in the center of the starting line, zigzagged down and back up between the cones, ran towards the last cone on the far side, and finished at the finish line. Participants performed two maximal trials with at least 10 minutes of rest in between. For statistical analysis, the fastest time between trials was taken and recorded [35].

### Statistical analysis

The statistical analysis and rain cloud graphs were performed using JASP 0.16.2 (JASP Team, 2018; https://jasp-stats.org/, accessed November 1, 2023). We checked the normal distribution of the data obtained using the Shapiro-Wilk test. The effect of PAPE (Wup and Wup+PAPE) on RT scores (visual, auditory, and mixed) was analyzed separately for each value by the Wilcoxon signed-rank test. The effect of PAPE (Wup and Wup+PAPE) on COD scores was analyzed by the Wilcoxon signed-rank test. For all statistical tests, the significance level was set at α <  0.05, and effect sizes were calculated using rank biserial (r_B_) on JASP. The rank biserial correlations are rated as follows: < 0.1: very small effect; 0.1: small effect; 0.3: medium effect; 0.5: large effect [36].

## Results

The Wilcoxon signed-rank test was conducted to compare visual RT between the Wup and Wup+PAPE conditions. The results indicated a significant improvement in visual RT in the Wup+PAPE condition (Mdn =  270 ms) compared to the Wup condition (Mdn =  305 ms), W =  65, *p* <  0.005. This demonstrates that the Wup+PAPE protocol led to a statistically significant reduction in visual RT. With a rank-biserial correlation of rB =  0.97, the effect size was classified as large, reinforcing the substantial impact of the Wup+PAPE protocol on visual RT.

**Fig 2:**
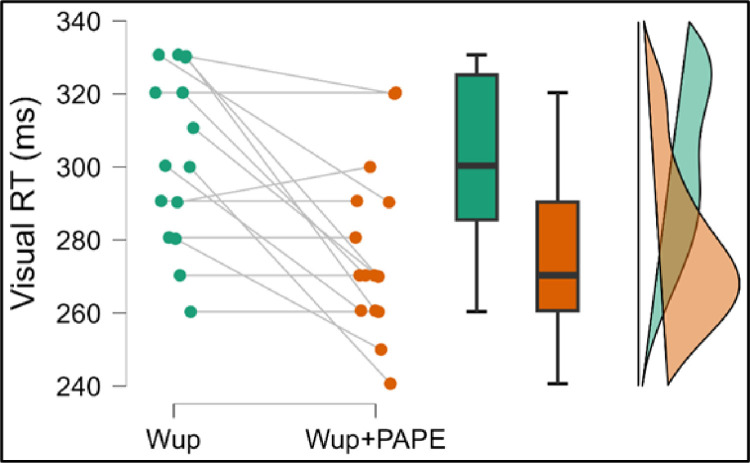
Within group visual RT comparisons Wup and Wup+PAPE # denotes statistically significant different within group comparisons, p <  0.05.

Fig 2 illustrates the comparison of visual RT across both conditions, showing a clear reduction in reaction times when Wup+PAPE was incorporated.

**Fig 3:**
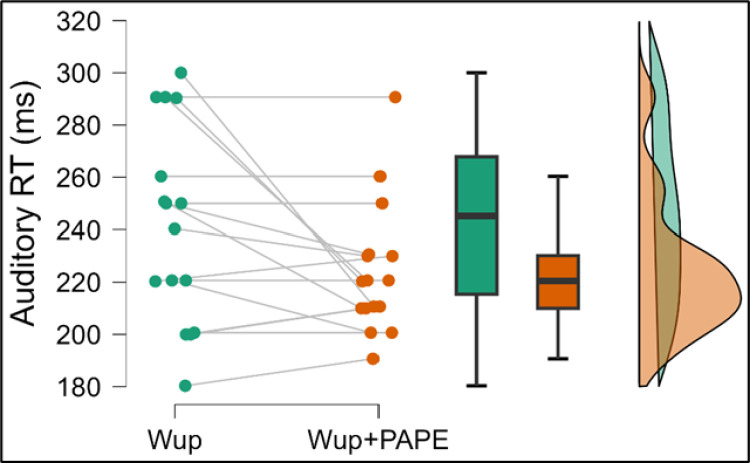
Within group auditory RT comparisons Wup and Wup+PAPE # denotes statistically significant different within group comparisons, p <  0.05.

The Wilcoxon signed-rank test was performed to compare auditory RT between the Wup and Wup+PAPE conditions. No significant difference was found in auditory RT between the two conditions, with the Wup condition (Mdn =  245 ms) showing slightly faster reaction times than the Wup+PAPE condition (Mdn =  220 ms), W =  52, *p* =  0.068. Although the result did not reach statistical significance, the effect size indicated by the rank-biserial correlation (rB =  0.63) was considered large, suggesting a notable practical difference despite the p-value. Fig 3 shows the auditory RT values in both conditions.

A Wilcoxon signed-rank test was used to assess the difference in mixed RT between the Wup and Wup+PAPE conditions. The analysis revealed a statistically significant reduction in mixed RT in the Wup+PAPE condition (Mdn =  275 ms) compared to the Wup condition (Mdn =  315 ms), W =  61, *p* =  0.013. This result indicates that the PAPE intervention had a meaningful effect on improving mixed RT.

**Fig 4:**
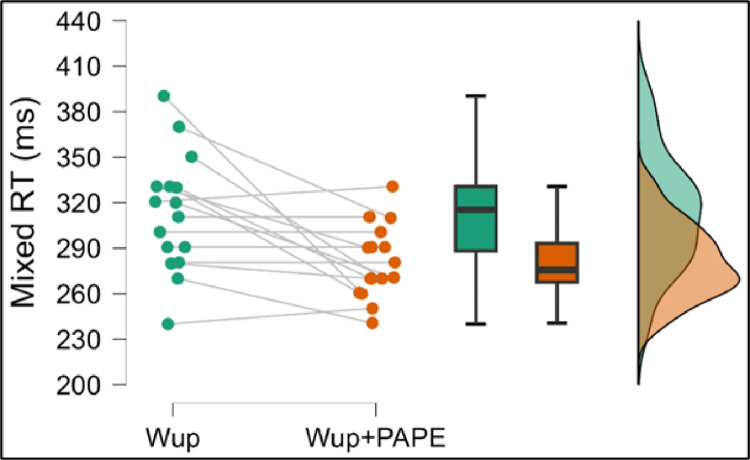
Within group mixed RT comparisons Wup and Wup+PAPE # denotes statistically significant different within group comparisons, p <  0.05.

The rank-biserial correlation (rB =  0.86) suggested a large effect size, confirming that the observed improvement in mixed RT was not only statistically significant but also practically relevant. Fig 4 shows the mixed RT values in both conditions.

The Wilcoxon signed-rank test was conducted to compare change of direction (COD) times between the Wup and Wup+PAPE conditions. The analysis revealed a significant improvement in COD times for the Wup+PAPE condition (Mdn =  15.49 sec) compared to the Wup condition (Mdn =  15.96 sec), W =  120, *p* <  0.001. This result highlights a statistically significant difference between the two conditions.

**Fig 5:**
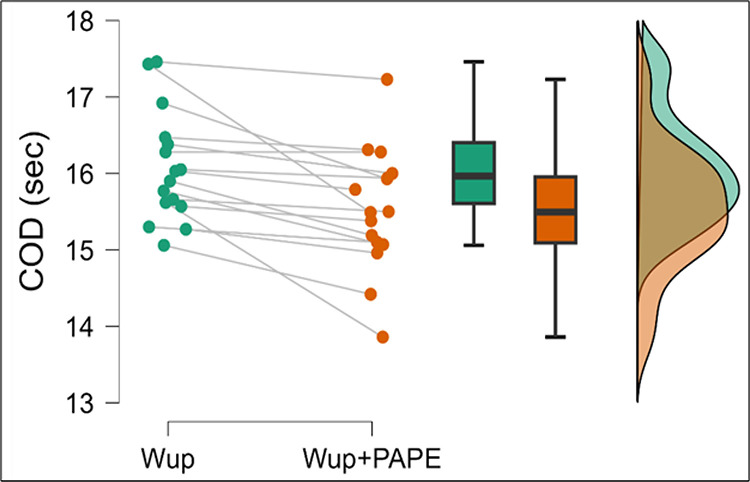
Within group COD comparisons Wup and Wup+PAPE. # denotes statistically significant different within group comparisons, p <  0.05.

The rank-biserial correlation (rB =  1.00) indicated a large effect size, suggesting that the addition of the PAPE protocol had a substantial impact on improving COD performance. Fig 5 presents the COD values in both conditions. The comparison between Wup condition and Wup+PAPE condition is presented in Table 1.

**Table 1:** Comparison between the Wup condition with the Wup+PAPE condition.

	Wup Mean ± SD	Wup + PAPE Mean ± SD	∆	*p* value	Effect Size (rB)
Visual RT (ms)	305.41 ± 24.79	276.58 ± 22.69	−27.83	.005 *	0.97
Auditory RT (ms)	241.60 ± 37.76	224.16 ± 25.21	−17.44	.068	0.63
Mixed RT (ms)	312.87 ± 38.39	281.04 ± 24.13	−31.83	.013 *	0.86
Change of Direction (sec)	16.07 ± 0.72	15.52 ± 0.79	−0.55	.001 *	1.00

## Discussion

The aim of this study was to investigate the effect of PAPE intervention induced by 80% 1RM back squat exercise on COD and RT in basketball players. The main findings of this study support our hypothesis that PAPE intervention significantly improved COD performances (p < 0.001). However, PAPE significantly improved visual RT (p < 0.005) performance and relatively non-significantly improved auditory RT (p < 0.068) performance, partially supporting our second hypothesis.

We believe that this is the first study in literature to investigate the effect of PAPE intervention on visual, auditory, and mixed RT. As a result of the study, significant improvement in visual RT and relative improvement in auditory stimuli were observed. In the literature, there are many research results showing that time-related cognitive tasks improve in exercise-based studies [37,38]. When compared with the mean values of the groups who did not exercise with the same measurement method in the literature, the mean values of visual and auditory RT of the basketball player participants in this study are compatible with chronic exercise adaptations. Although the baseline level, especially auditory RT improved relatively, it is thought that it is not significant due to chronic adaptation. Accordingly, a significant improvement in RT was reported after high-intensity acute resistance exercise (80% 1RM x5 repetitions) [11]. Similarly, a study examining changes in cognitive function using a computer-based Stroop test program 15 minutes after high-intensity resistance consisting of 8-10 rep. at 80% 1RM showed a significantly greater improvement in RT [39]. Another study reported that a single maximum load (100% of 10RM) resistance exercise resulted in faster RT for the Stroop task 15 minutes after exercise cessation [40]. Differences in these improvements are thought to be related to differences in the type of stimulus and the time it takes to reach the brain. The basic steps of the reaction function include: stimulus reaching the sensory organ, transduction into a neural signal by the sensory organ, neural transmissions, processing and muscle activation [41]. In the literature examined, it takes 8-10 ms for an auditory stimulus to reach the brain, while a visual stimulus takes 20-40 ms [42]. This was associated with the number of synapses in the visual pathway compared to the auditory pathway [43]. Shelton and Kumar [44] emphasize that the auditory response time may be faster than the visual response time since auditory stimuli reach the cortex faster than visual stimuli. As a result, the faster the stimulus reaches the brain, the faster the signal is processed, and the responses are sent for the required motor response. It is difficult to predict which steps of these processes were improved as a result of the study. It is difficult to interpret whether this improvement is due to the brain-based organization of the reaction or the physiological mechanisms of the motor response that occur through central mechanisms. To the best of our knowledge, motor phases (approximately 80 ms) in reaction measurements take place in a shorter time than other processes. Speculatively, it is thought that increased peripheral lactate response may be a possible factor in the warm-up protocol performed with PAPE intervention in our study. Lactate is a source that can pass through the brain-blood barrier and can be used as energy, especially by astrocytes [45]. Furthermore, lactate, a precursor, triggers the glutamate-glutamine reaction. This neurochemical exchange has the effect of enhancing communication and coordination between neurons [46]. In the process, lactate may produce a potentiation effect in the brain for a duration [47]. In addition, from the perspective of the neuroendocrine hypothesis [48], this high-intensity intervention with 5 repetitions for stimulation only may have positively affected the reaction tasks. There is a need to investigate the issue with more detailed experimental designs in future research.

Secondarily, in muscle fibrils with dense nerve fibers such as type 2 in peripheral processes, a PAPE-induced stimulation may also increase the amount of messengers such as acetylcholine in motor pathways [49]. The lifting technique and the extra motivation and concentration needed during the PAPE attempt may also have had a positive impact on the processes [50]. These findings demonstrated that the resistance-induced PAPE protocol is an effective paradigm for eliciting acute exercise-induced changes in various cognitive features. Our reaction findings support that PAPE may contribute more positively to visual stimuli. Since rapid responses to visual stimuli in basketball have the potential to affect the outcome, it is thought that PAPE intervention should be applied in the warm-up models of basketball players.

Considering the COD findings obtained in the study, it was found that PAPE intervention (80% 1RM x5 reps.) increased COD performance. The Illinois agility test, which was preferred in the study, is a model with high neuromuscular load COD. In addition, this model is similar to the agility requirements of basketball used on the court. Some studies in the literature have evaluated the acute effects of resistance exercise on agility performance. It was reported that 85% 1RMx5 repetitions of the back squat PAPE intervention significantly improved the modified agility the t-test result in handball players [16]. In the other study, in volleyball players, t-agility test improved significantly after 80% 1RMx5 repetitions of back squat compared to the baseline condition [51]. Another study reported that PAPE intervention with 85% 1RMx3 leg press improved the modified 5-0-5 agility test in tennis players [52]. In contrast, it was reported that there was no change in agility (5-0-5) performance time following barbell hip thrusts (85% 1RM) compared to the control condition in college-aged men and women [53]. In a study conducted with court sports players, it was observed that 50% 1RMx5, 60% 1RMx3, 90% 1RMx3 parallel back squats improved agility shuttle test results compared to control [54]. In addition to these improvements seen in the literature in tests that structurally involve linear accelerations such as the t-test, our study showed that the ıllinois agility test, which we think is more suitable for use in branches that involve more directional tasks such as acceleration and deceleration, also improved. During acceleration-deceleration and turns, neuromuscular workload increases at a high level [55]. Resistance-based PAPE intervention applied in 80% of 1RM positively affects neuromuscular output [31,32]. We can only make hypothetical comments about the potential muscular and neural mechanisms of action of PAPE intervention leading to higher test performance. Based on our data and past studies support that, resistance-based PAPE stimulus (maximal or near maximal) causes greater motor unit excitability, potentially benefiting actions related to motor unit synchronization or motor unit enhanced central input, and may have increased motor unit excitability [2]. In addition, it is thought that the information obtained in our study may shed light on similar motor tasks in real field conditions.

## Conclusion

The results showed that PAPE intervention with resistance-based back squat is a usable method for pre-match preparation of reaction and COD characteristics in male basketball players. The similar improvement in COD performance in within-group evaluations indicates that the applied load can be used as a reference load, while within-group individual differences in visual auditory and mix reaction draw attention to the need to determine an individual-specific PAPE load. Physiologically, the high motor unit engagement and firing required for COD was provided by the PAPE load. We think that the PAPE intervention method we applied in all sports branches where the need for COD is high can be used by practitioners.

Reaction time is among the cognitive needs specific to the game of basketball and has positive effects on processes such as decision-making and attention. Especially visual reaction is an important cognitive need for in-game actions. The significant improvement in visual reaction time obtained in the study showed that the PAPE intervention we applied may also help cognitive preparation for the match/game.

On the other hand, new study designs are needed to answer the question of how long both traits are positively affected by PAPE intervention. In addition, it is noteworthy that the effects of variables such as gender difference
